# *Rickettsia parkeri* Rickettsiosis Resembling Sweet Syndrome: A Differential Diagnosis for Critical Discussion

**DOI:** 10.3390/idr17030045

**Published:** 2025-05-01

**Authors:** Lucas S. Blanton, Sarah E. Muir, Nicole L. Mendell, David H. Walker

**Affiliations:** 1 Department of Internal Medicine—Division of Infectious Diseases, University of Texas Medical Branch, Galveston, TX 77555, USA; 2 John Sealy School of Medicine, University of Texas Medical Branch, Galveston, TX 77555, USA; semuir@utmb.edu; 3 Department of Pathology, University of Texas Medical Branch, Galveston, TX 77555, USA; nlmendel@utmb.edu (N.L.M.); dwalker@utmb.edu (D.H.W.)

**Keywords:** spotted fever group rickettsiosis, *Rickettsia parkeri*, Sweet syndrome, immunohistochemistry

## Abstract

**Introduction:** Spotted fever group (SFG) rickettsioses are tick-transmitted infections caused by Gram-negative, obligately intracellular bacteria in the genus *Rickettsia*. They present as an acute undifferentiated febrile illness, and they are often accompanied by rash and/or eschar. Although the rash of SFG rickettsioses usually consists of macules and papules, some, like in *Rickettsia parkeri* rickettsiosis, can also manifest with papulovesicular or pustular lesions. **Case:** We herein present a case of SFG rickettsiosis, due to *R. parkeri*, that masqueraded as Sweet syndrome (the prototype neutrophilic dermatosis) after the initial results of a shave biopsy. Further investigation of the biopsy specimen by immunohistochemical and PCR analysis would eventually confirm SFG rickettsiosis, with *R. parkeri* being detected by real-time PCR. **Discussion:** *Rickettsia parkeri* is transmitted by the Gulf Coast tick (*Amblyomma maculatum*) and is an increasingly recognized cause of SFG rickettsiosis in the United States. *Rickettsia parkeri* should be considered in those with an acute undifferentiated febrile illness with lesions that are pustular or papulovesicular, as prompt recognition and empirical administration of doxycycline results in the rapid resolution of symptoms.

## 1. Introduction

Spotted fever group (SFG) rickettsioses are tick-transmitted infections caused by Gram-negative, obligately intracellular bacteria in the genus *Rickettsia*. The SFG comprises over 20 named species distributed worldwide [1]. Pathogenic members of the SFG cause an acute undifferentiated febrile illness with varying degrees of severity. In the Americas, *R. rickettsii* causes Rocky Mountain spotted fever (RMSF)—a severe life-threatening rickettsiosis with a case fatality rate upwards of 40% [2]. In juxtaposition, *R. parkeri* causes a much milder disease, with no reported deaths attributed to this species [3,4,5]. Rickettsioses are often accompanied by cutaneous manifestations, such as rash and/or eschar. Sweet syndrome, also called acute febrile neutrophilic dermatosis, is the prototype neutrophilic dermatosis (a group of heterogeneous diseases characterized pathologically by diffuse and perivascular neutrophilic infiltrates). In addition to Sweet syndrome, the neutrophilic dermatoses include the following: pyoderma gangrenosum; Behçet disease; bowel-associated dermatosis–arthritis syndrome; synovitis, acne, pustulosis, hyperostosis, and osteitis (SAPHO) syndrome; and erosive pustular dermatosis [6]. Cutaneous manifestations of Sweet syndrome include tender and/or erythematous plaques, nodules, papules, and vesicles [7] (the latter two being features of some SFG rickettsioses) [3]. The diagnosis of Sweet syndrome is guided by a set of major and minor diagnostic criteria. Major criteria include the abrupt onset of typical cutaneous lesions and consistent histopathology (usually, a diffuse nodular and perivascular, predominantly neutrophilic, dermal infiltrate). Minor criteria include preceding fever or infection; accompanying fever, arthralgia, conjunctivitis, or underlying malignant lesion; leukocytosis; response to systemic corticosteroids and not to antibiotics; and increased erythrocyte sedimentation rate. The presence of both major and at least two minor criteria helps establish the diagnosis [6]. Skin biopsy specimens from those with SFG rickettsioses can occasionally share pathologic features with neutrophilic dermatoses. Recognizing signs, symptoms, and epidemiologic risk factors consistent with a rickettsiosis is imperative to empirically starting doxycycline, which quickly abates symptoms. We herein report a case of SFG rickettsiosis, likely due to *Rickettsia parkeri*, masquerading as Sweet syndrome.

## 2. Case Presentation

A 60-year-old healthy woman developed an acute febrile illness in the spring of 2023. Her symptoms started on April 16 when she first noted a painless, non-pruritic, circular erythematous patch (1 × 1 cm) on her right thigh. Two days later, she developed subjective fever and chills. On 20 April, the woman visited her family physician, and the erythematous patch on her right thigh had enlarged (4 × 4 cm) and was associated with a central papule. A cutaneous staphylococcal infection was suspected. She was therefore prescribed a 10-day course of trimethoprim-sulfamethoxazole (TMP-SMX) 160/800 mg oral twice daily and 2% mupirocin ointment. Despite this treatment, the woman’s fever intensified (up to 39 °C) in the following days. Her fever was accompanied by headache, fatigue, malaise, and arthralgia. On 22 April, the woman developed scattered 2–5 mm erythematous macules over her trunk, which later spread to her extremities. She sought care with a dermatologist on 24 April. Two 5 mm diameter shave biopsies were performed (both of papular lesions on the upper right thigh). Blood was also collected for laboratory analysis.

The patient was previously in excellent health, walking approximately 7 km daily, and was free of chronic medical conditions. She lived in a wooded area north of Houston, Texas. She noted seeing opossums, raccoons, rats, and various other wildlife around her neighborhood, but she denied close contact. She owned a dog that roamed in and out of the wooded area adjacent to her home, but the dog received regular treatments to prevent flea and tick infestation. On 13 April, approximately 3 days before illness, she visited the coastal city of Galveston, Texas (approximately 140 km southeast of her home), but otherwise denied recent travel. She did not recall any preceding tick or flea bites, although she performed yard work in the days prior to her illness.

Laboratory testing performed on the day of evaluation by her dermatologist (24 April) revealed a leukocyte count of 2700 cells/mm^3^ (normal range 3800–10,800 cells/mm^3^), platelet count of 97,000/mm^3^ (normal range 140,000–400,000/mm^3^), and serum sodium of 130 mmol/L (normal range 135–136 mmol/L). All other values on the hemogram and metabolic panel (including hepatic transaminases) were normal.

These laboratory abnormalities (leukopenia, thrombocytopenia, and hyponatremia), when their results returned the next day from the laboratory, prompted her dermatologist to consider murine typhus (an infection endemic in coastal Texas) as a cause of her fever and rash. TMP-SMX was discontinued, and doxycycline 100 mg oral twice daily was initiated on 25 April. The patient returned to her usual state of health within a few days with resolution of fever, cutaneous lesions, and other associated symptoms, reinforcing the initial presumptive diagnosis of murine typhus.

**Figure 1:**
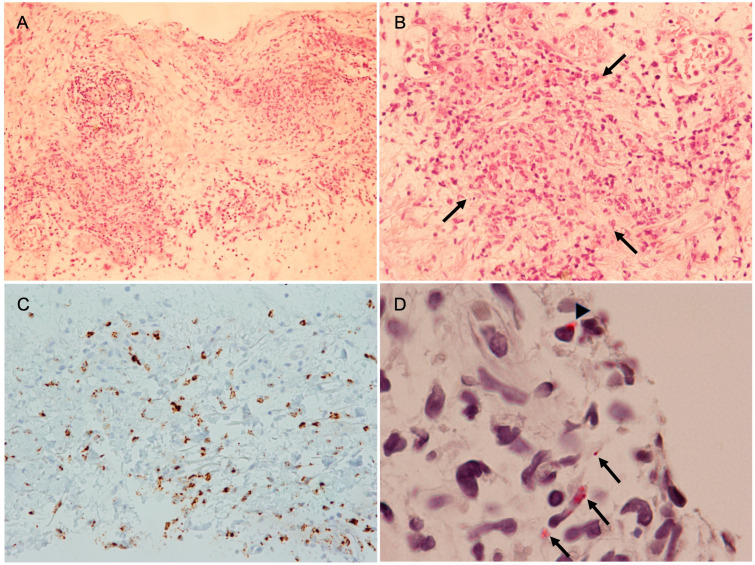
Photomicrographs of a shave skin biopsy obtained from a papular lesion. (**A**) Low-power photomicrograph showing the architecture of epidermal and superficial dermal layers (hematoxylin and eosin stain; ×100). (**B**) Intense perivasculitis is noted in the area contained by the arrows (hematoxylin and eosin stain; ×200). (**C**) Numerous myeloperoxidase-stained cells are demonstrated, indicating neutrophilic infiltrates (myeloperoxidase stain; ×200). (**D**) Immunohistochemical staining of the shave biopsy specimen for spotted fever group rickettsiae demonstrates infection of longitudinally arranged endothelial cells (arrows) and the presence of cytosolic rickettsiae (arrowhead) (×1000).

Following her recovery, dermatopathologic results of the shave biopsies became available and demonstrated spongiosis of the epidermis, marked papillary dermal edema, dense leukocytoclastic neutrophilic infiltrate, and numerous myeloperoxidase staining cells forming a neutrophilic dermatosis (Figure 1). These findings were interpreted as being consistent with the histopathology associated with Sweet syndrome.

Eager to clarify her underlying diagnosis, the patient requested additional testing. At a follow-up visit with her dermatologist, convalescent serum (obtained on 11 May [approximately 25 days after the onset of illness]) was sent to a commercial laboratory for serologic testing. An indirect immunofluorescence assay (IFA) for SFG rickettsiae demonstrated IgG reactivity with a titer of ≥1:256. Typhus group rickettsial IFA, *Ehrlichia chaffeensis* IFA, *Borrelia burgdorferi* immunoblots (IgM and IgG), and *Coxiella burnettii* IFA (phase I and II) were all non-reactive. The reactive SFG rickettsial IFA test prompted the patient to request that the formalin-fixed paraffin-embedded (FFPE) skin biopsies be sent to the University of Texas Medical Branch for further testing. Immunohistochemical testing for SFG organisms was performed as previously described [8] with modification of the primary antibody (rabbit polyclonal anti-*R. conorii* Malish 7 strain at a dilution of 1:500). Immunohistochemistry revealed the presence of SFG rickettsiae within dermal endothelium (Figure 1).

To identify the causative SFG species, DNA was extracted from cut sections of the FFPE shave biopsy specimens [8] and multiplexed probe-based real-time PCR was performed, as described by Gaines and colleagues [9]. *Rickettsia parkeri* DNA was amplified at a mean quantification cycle of 33.3 from the specimen that was tested in triplicate.

## 3. Discussion

The case presented describes confirmed SFG rickettsiosis, likely caused by *R. parkeri*, masquerading as Sweet syndrome. Sweet syndrome was first described in 1964 [10] and is the prototype neutrophilic dermatosis, sharing overlapping histologic features with other neutrophilic dermatoses (e.g., pyoderma gangrenosum, Behçet disease, and bowel-associated dermatosis–arthritis syndrome) [6]. The diagnosis of Sweet syndrome is guided by a set of major and minor diagnostic criteria. The major criteria include the abrupt onset of typical cutaneous lesions (tender or painful erythematous/violaceous plaques or nodules) and consistent histopathology. The histologic presentation is usually a diffuse nodular and perivascular, predominantly neutrophilic, dermal infiltrate. Although vasculitis is usually not present, leukocytoclastic vasculitis is occasionally described. Minor criteria include preceding fever or infection; accompanying fever, arthralgia, conjunctivitis, or underlying malignant lesion; leukocytosis; response to systemic corticosteroids and not to antibiotics; and an increased erythrocyte sedimentation rate. The presence of both major and at least two minor criteria helps establish the diagnosis [11]. The pathologic findings of Sweet syndrome may occur deeper within the dermis and even the subcutis [6], so a punch biopsy, rather than a shave biopsy as obtained in this case, is usually preferred.

Sweet syndrome has been associated with a variety of disorders or triggers, including malignancies, medications, inflammatory bowel disease, other autoimmune disorders (e.g., systemic lupus erythematosus, rheumatoid arthritis, dermatomyositis, and sarcoidosis), and infection [6,7,12]. Infection is an inflammatory trigger that occurs in the weeks prior to the onset of Sweet syndrome, rather than in the setting of acute infection. In a well-described case series, Sweet syndrome was associated with infection in 23% of cases, but these infections (usually viral respiratory syndromes) occurred several weeks prior to the onset of characteristic dermatologic findings [7]. In addition to upper respiratory tract infections, infectious triggers include other viruses (cytomegalovirus, HIV, HCV, and HBV), bacteria (streptococci and *Yersinia*), fungi (*Sporothrix* and *Coccidioides*), and mycobacteria (*Mycobacterium tuberculosis*, *M. leprae*, and nontuberculous mycobacteria) [6]. Although the underlying pathogenesis is unknown, it is hypothesized that cytokine dysregulation—especially granulocyte colony-stimulating factor—plays a contributing role [6,13,14].

The various diagnostic criteria of Sweet syndrome have overlap with a variety of other syndromes. When evaluating one with suspected Sweet syndrome, it is important to critically consider other entities in the differential diagnosis. The case presented in this report does indeed match many of these features, but when considering the clinical picture as a whole, the diagnostic criteria of Sweet syndrome are not adequately met. The patient did have erythematous papules, but they were not painful or pruritic. She also had an erythematous patch early during her presentation, but plaques, rather than patches, are a typical cutaneous manifestation. The patient’s fever was eventually attributed to infection, but, as outlined above, infectious triggers occur in the weeks prior to the development of Sweet syndrome, rather than occurring simultaneously or after a very short interval [6,7]. The patient also responded quickly and completely to an antibiotic (doxycycline) rather than corticosteroids. In this case, during the early stages of her workup, it seems that the provisional diagnosis of Sweet syndrome was disproportionally based on pathologic findings. As outlined in the aforementioned criteria for considering the diagnosis of Sweet syndrome, more than histopathological findings must be considered.

SFG *Rickettsia* species are Gram-negative, obligately intracellular coccobacilli transmitted by ticks. In the United States, disease-causing SFG rickettsiae include *R. rickettsii*, *R. parkeri*, and *R. rickettsii* subspecies *californica* (previously known as *Rickettsia* sp. 364D) [1,15,16]. *Rickettsia typhi* (the causative flea-borne agent of murine typhus) [17] and *R. akari* (the causative mite-borne agent of rickettsialpox) [18] are also endemic to the U.S. but belong to different phylogenetic groups to SFG rickettsiae (typhus and transitional groups, respectively) [19]. Symptoms of rickettsioses are undifferentiated and usually include fever, headache, and myalgia. A macular and/or papular rash is a frequent sign. Thrombocytopenia, elevated hepatic transaminases, and hyponatremia are often noted on routine laboratory testing [20]. Although not a feature of RMSF, most SFG rickettsioses are associated with an eschar (crusty, often black, area of cutaneous necrosis) at the tick bite site [1]. Confirmatory diagnostic testing for SFG rickettsioses includes seroconversion or a four-fold increase in IgG-specific antibody titer against SFG rickettsiae; immunohistochemical detection of SFG antigen within biopsied tissue; detection of SFG rickettsial nucleic acid from a clinical specimen; or culture isolation (rarely undertaken due to the need for cell culture techniques and use of a biosafety level-3 facility) [15]. Due to the cross-reactivity of antibodies stimulated by the infecting SFG agent to other SFG rickettsiae, a species-specific diagnosis is not possible using serology or immunohistochemistry. A diagnosis at the species level is only possible via the sequencing of PCR products or the use of species-specific primers and probes [21].

*Rickettsia parkeri* is endemic to the southeast and Gulf Coast U.S. [1], where its primary tick vector, *Amblyomma maculatum*, is distributed [22]. Recently, a northward expansion of *A. maculatum* has been reported in proximity to densely populated areas—a possible result of climate change, grassland reclamation, and changing patterns of migratory birds [23]. *Rickettsia parkeri* causes an eschar-associated SFG rickettsiosis (eschar found in 91% of confirmed cases) [5]. In addition to eschar and the typical erythematous macules and/or papules associated with other rickettsioses, *R. parkeri* infection can also manifest with papulovesicular or pustular lesions [3,5]. These lesions are also seen in those with rickettsialpox and African tick bite fever. Caused by *Rickettsia africae*, an organism phylogenetically similar to *R. parkeri*, African tick bite fever is a common cause of fever in travelers to sub-Saharan Africa [24]. Compared to RMSF, *R. parkeri* rickettsiosis is relatively mild, with no reported deaths [3,5]. In the case presented, the physical resemblance of the skin lesions, histopathology, and systemic symptoms originally suggested Sweet syndrome, but active infection, demonstrated by the observation of rickettsial antigen within the dermis by immunohistochemistry, the development of a significant antibody response to SFG rickettsiae, and the amplification of rickettsial DNA by PCR, as well as the swift resolution of systemic and cutaneous manifestations with doxycycline confirms SFG rickettsiosis as the cause of her illness. The PCR assay employing species-specific molecular probes supports *R. parkeri* as the causative infectious agent [15,25]. It should be noted that the patient was prescribed TMP-SMX early in her illness. This agent has been associated with greater disease severity in those infected with *Rickettsia* species [26,27]. It is unclear if the use of TMP-SMX had a negative impact on this woman’s disease course, as her illness was relatively mild and did not require hospitalization.

The classic histopathologic description of the rash of an SFG rickettsiosis (usually found in the center of a petechial macule or papule) is lymphohistiocytic vasculitis of the dermal blood vessels with extravasation of erythrocytes [28]. Lesions in those with *R. parkeri* rickettsiosis have occasionally been described as containing abundant neutrophils [3,29], and eschars of those with African tick bite fever demonstrate significantly more neutrophils than the eschars of Mediterranean spotted fever (a more severe disease caused by *Rickettsia conorii*) [30]. Interestingly, although rickettsialpox often presents with a prominent vesiculopustular rash, the histopathology of cutaneous lesions reveals few neutrophils [31]. A patient with Queensland tick typhus (*Rickettsia australis*) who experienced a complete response to doxycycline has also been reported to have a Sweet syndrome-like neutrophilic infiltrate on biopsy of a papular lesion [32].

## 4. Conclusions

SFG rickettsioses are tick-transmitted infections that present as an acute febrile illness often accompanied by cutaneous lesions, such as maculopapular rash, petechiae, and, often, an eschar. Some rickettsioses, such as *Rickettsia parkeri* rickettsiosis, African tick bite fever, and rickettsialpox, can present with pustular and papulovesicular lesions, which can be mistaken for other clinical entities. When histopathology demonstrates prominent neutrophilic infiltrates, rather than the lymphohistiocytic infiltrates more typical of a rickettsiosis, a patient may erroneously be diagnosed with Sweet syndrome. When considering a patient with possible Sweet syndrome, it is imperative to carefully consider epidemiologic features that may prioritize other entities on the differential diagnosis. Recognizing signs and symptoms compatible with SFG rickettsioses is paramount to the timely initiation of empiric doxycycline.

## Data Availability

No data sets were generated by the author for the purposes of this paper. Data presented in the context of the literature review have been previously published by others and cited within the manuscript.
